# Mode of Action of Farnesol, the “Noble Unknown” in Particular in Ca^2+^ Homeostasis, and Its Juvenile Hormone-Esters in Evolutionary Retrospect

**DOI:** 10.3389/fnins.2019.00141

**Published:** 2019-02-25

**Authors:** Arnold De Loof, Liliane Schoofs

**Affiliations:** Functional Genomics and Proteomics Group, Department of Biology, KU Leuven–University of Leuven, Leuven, Belgium

**Keywords:** JH receptor, prostate, male accessory glands, corpora allata, insect hormones, mevalonate, GPCR, Alzheimer

## Abstract

Farnesol, the sesquiterpenoid precursor of insect juvenile hormones (JH) that itself has JH activity, existed already long before animals and their hormones came into being. Although it is omnipresent in all eukaryotes, this molecule remains a “noble unknown” in cell physiology. It is neither documented as a hormone nor as another type of signaling molecule. To date, its function as an intermediate in the synthesis of squalene-cholesterol-steroids in chordates/vertebrates, and of the insect/arthropod JHs, esters of farnesol, in the mevalonate biosynthetic pathway is assumed to be the only one. This assumption neglects that already two decades ago, farnesol has been shown to be a potent endogenous inhibitor of N-type voltage-gated Ca^2+^ channels in some mammalian cell types. The tandem mevalonate pathway and Ca^2+^ channels originated early in eukaryotic evolution, and has since been well conserved, “promoting” it as a ubiquitous player in Ca^2+^ homeostasis in all eukaryotes. This paper accentuates how this drastic change in thinking gained momentum after the discovery by Paroulek and Sláma that the huge amounts of JH I in male accessory glands of the *Cecropia* moth, are actually synthesized in these glands themselves and not in the corpora allata, the hitherto assumed unique synthesis site of such compounds. In addition, MAG-JHs have no hormonal- but an *exocrine* function. Here we hypothesize that MAG-JHs may function in protecting the spermatozoa against toxic Ca^2+^ concentrations, and in enabling their flagellum to undulate. They may do so by acting through membrane receptors. Our novel paradigm assigns to farnesol/JHs a function of flexible hydrophobic molecular valves for restricting untimely Ca^2+^-passage through some types of canonical Ca^2+^channels, using covalently bound farnesyl- or geranyl-geranyl group attachment as well as GPCRs-G proteins all containing a prenyl group. The high rotatable bond count, and their horseshoe-shape are instrumental to their valve function. In our paradigm, Met/Tai and Gce, to date generally thought to be *the* (only) functional (nuclear) receptors for JHs, are classified as probable Ca^2+^-sensitive transcription factors. Some theoretical and practical considerations for possible applications in a medical context will be discussed.

## Introduction

When the chemical nature of insect juvenile hormone was identified in 1968 by Röller and Dahm, the majority of the nowadays well-developed subdisciplines in endocrinology and evolutionary theory were still in their infancy. Comparing the endocrine system of insects with that of vertebrates was not yet an acceptable research strategy to many endocrinologists. Endocrine archaeology did not exist. The major focus was on uncovering its mode of action, a challenge that to date still has not yet met full success. When dealing with the mode of action of either juvenile hormone (JH) and/or ecdysteroids, the most accepted model focuses almost exclusively on the role of nuclear receptors and/or transcription factors, thereby minimalizing a possible role for membrane receptors (Jindra et al., 2013, 2015a,b; Lenaerts et al., 2018). This neglects basic knowledge in endocrinology that says that, perhaps apart from gaseous hormones (e.g., gassing with ethylene speeds up fruit ripening), no hormone can act somewhere in the cell, e.g., in the nucleus, without starting signaling at the level of the plasma membrane first. Do both key insect hormones form an exception? Or has something important been unintentionally or deliberately overlooked, and put away as “of no physiological importance”? Two events in particular were causal to putting some insect endocrinologists on the wrong track.

The first event was the misgiven name to 10,11-epoxyhomofarnesoate ester, (IUPAC name: methyl (2E,6E)-7-ethyl-9-[(2R,3S)-3-ethyl-3-methyloxiran-2-yl]-3-methylnona-2,6-dienoate), namely “Cecropia juvenile hormone I (JH I)” or “C18 JH.” This compound had been extracted from male accessory glands (MAGs) (the equivalent of the mammalian prostate) of adult males of the moth *Hyalophora cecropia*, where it does not display any known hormonal function. It nevertheless was denominated “hormone” because it was highly active in typical bioassays where the hormonal activity of this compound could be demonstrated. The exact role of *exocrine* non-hormonal “MAG-JH I” (a contradictory wording) in the male reproductive system has so far not yet been clarified. However, the finding that farnesol, the precursor of all JH-isomers and itself a compound with moderate juvenile hormone activity (Wigglesworth, 1969), acts as a selective inhibitor of voltage-gated Ca^2+^ channels (Luft et al., 1999; Roullet et al., 1999; §5.3) may lead to a plausible explanation. These studies state that the tandem Farnesol-Voltage-gated Ca^2+^ channels, which was already operational long before animals even came into being, and which has been very well conserved during evolution up to this day, may already have served at least three key functions in a unicellular choanoflagellate-type organism. Exactly such eukaryote organism is currently thought to have been ancestral to sponges first, and later in evolution, to all other animals as well (Cavalier-Smith, 2017). These functions may have been: (1) acting as a flexible molecular valve for restricting the gating in helix-bundle transmembrane proteins, e.g., selected ion channels and G-Protein Coupled receptors (GPCRs); (2) control of the undulating movement of the flagellum with functions in feeding and, if free living, in locomotion; (3) concurrently, protection against Ca^2+^-induced cell death. Some of the mechanisms instrumental to such functions were probably inherited from even more ancient ancestors, both prokaryotic and eukaryotic. Needless to mention that unicellular organisms do not make use of hormones.

The second cause of confusion was that 50 years ago insect endocrinologists preferred the model of Karlson and Sekeris (1966) over other, also scientifically sound models. This model stated that ecdysteroids, in particular 20-hydroxyecdysone (20E) that is water-soluble, can enter the cell by simple diffusion through a lipophilic membrane (which is impossible), travel to the nucleus and finally control there the activity of selected genes. For lack of a better alternative, this model was intuitively extrapolated and adopted as a valid working hypothesis for JH as well. Thus the current nuclear receptor models for both 20E and JH date back to the late 1960ties: they did not change much since, and they have not yet advanced a solution for coping with the hydrophilic-hydrophobic barriers for entering and leaving the lipid plasma membrane. But neither the hydrophilic steroid 20E, nor the lipophilic sesquiterpenoid JH can end up in the cytoplasm of any cell type by simple diffusion. To exert all their functions they have to use specific *membrane receptors* of which some reside in the plasma membrane, others in intracellular membranes.

The already identified *membrane receptors* for farnesol/farnesol-like substances (FLS: JHs) and 20E involve the Ca^2+^-homeostasis systems of the target cells. Although it is such a basic fact in cell biology that Ca^2+^ is a ubiquitous intracellular messenger, which encodes information by temporal and spatial patterns of concentration (Jimenez-Gonzalez et al., 2006), the mechanism of Ca^2+^-induced release of Ca^2+^ from intracellular Ca^2+^ storage sites resulting from hormonal stimulation is not yet a generally accepted way of thinking in insect endocrinology. Time has come to abandon the exclusivity of “the nuclear mode of action of JH and 20E controls all” way of thinking that is omnipresent in today’s insect endocrinology. MicroRNA also plays a role (Qu et al., 2018). A switch to an upgraded paradigm in which a more prominent role is attributed to *the integrated Ca^2+^-homeostasis system as being the primordial receptor system for both JH and ecdysteroids* seems to be unavoidable. It also urges for rethinking and upgrading the role of the mevalonate biosynthetic pathway and in particular of farnesol, an undeservedly “noble unknown” in vertebrate physiology and endocrinology, both in basic research and in possible practical applications e.g., in Alzheimer’s and other diseases.

## Reasons Why Farnesol is a “Noble Unknown”

Farnesol does not have the status of a hormone in vertebrates, neither of a control agent of Ca^2+^-homeostasis, but is only known as an intermediate in the mevalonate biosynthetic pathway that leads to the synthesis of cholesterol and steroids (§6.1). A first reason is that one did not consider the possibility that a so called “intermediate compound” in the biosynthesis of cholesterol and steroid hormones in vertebrates/mammals might have an important additional function on its own in Ca^2+^-homeostasis, in particular as a tool to restrict the gating of some types of Ca^2+^-channels (Roullet et al., 1999). Second, the notion “inbrome” that denotes compounds that after having inserted themselves into the lipid bilayer part of a membrane, could signal when they encounter a matching binding site on a membrane protein is rather recent (De Loof et al., 2015a). An inbrome can be delivered into the membrane from within the cell, and thus there is no need for transport in the bloodstream as a hormone. Third, farnesol is well documented as the precursor of juvenile hormones in insects, which are but esters of farnesol. However, chordates/vertebrates are generally thought to have no juvenilizing hormone of whatever type. Hence, the possibility that this view is wrong and that farnesol could nonetheless be involved – but not as a hormone - in realizing the juvenile state of vertebrates has only been advanced recently (De Loof et al., 2015a). Fourth, in contrast to the situation in insects with complete metamorphosis, there is no developmental stage in which farnesol/FLS disappears completely from the body in hemimetabolous insects and in chordates. *It is such fall to zero of the FLS titre* that induces drastic changes in the physiology of insects that can be exploited to design bioassays for detecting FLS-JH activity. Fifth, the study of endogenous sesquiterpenoids (farnesol/FLS) requires classical chemical chromatography, mass spectrometry and electrophysiology in addition to the molecular biological tools that are nowadays omnipresent in almost every life science laboratory. This list is not exhaustive. As a result, farnesol/FLS is *indeed a “noble unknown” in vertebrate endocrinology as cited in the Introduction.*

## Identifying a First Main Problem: the Huge Ca^2+^-Gradient Over Leaky Membranes and Its Consequences

**FIGURE 1:**
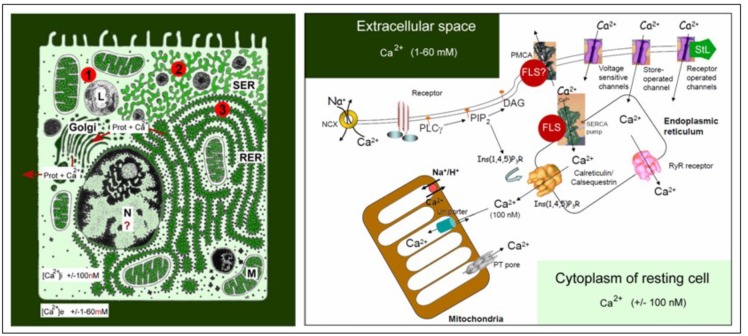
Schematic representation of the main Ca^2+^ gradients in eukaryotic animal cells (left panel) and of the main players in Ca^2+^-homeostasis (right panel). Left: Schematic representation of the Ca^2+^ gradient (adapted from De Loof, 2015, 2017: Copyright permission not required). The different shades of green are not meant to give an exact representation of differences in Ca^2+^-concentration. L, lysosome, N, nucleus; M, mitochondrion; RER, rough endoplasmic reticulum; SER, smooth endoplasmic reticulum. The red dots with 1, 2, and 3 correspond to the main mechanisms for keeping [Ca^2+^]i low. (1) Little influx of Ca^2+^ through the plasma membrane that can be countered by the activity of Ca^2+^-ATPases in the plasma membrane (PMCAs); (2) more influx and role for temporary storage of Ca^2+^ in membrane-limited organelles, in particular the SER; (3) high influx of Ca^2+^ triggers the removal of excess Ca^2+^ through the secretion of Ca^2+^-binding/transporting proteins via the RER. From De Loof (2017). Right: The major actors in the Ca^2+^-homeostasis system (slightly modified after Orrenius et al., 2003). The long legend as originally formulated by Orrenius et al. (2003) is not repeated here. It can be consulted in the Open Access paper by De Loof (2017). With thanks for the copyright permission for using the original figure and the legend from the publisher (Nature) and from Prof. S. Orrenius, both granted in 2014.

The link between farnesol and Ca^2+^-homeostasis, an important issue in our model, requires some background information. Ca^2+^ is the most abundant toxic pollutant on earth. Its concentration in the extracellular aquatic environment of cells, blood e.g., amounts to about 2 millimolar on the average versus about 100 nanomolar in the cytoplasm of unstimulated/resting cells. This represents a gradient of about 20,000 fold. As a result, the passive entry of excess Ca^2+^ into the cytoplasm through channels in the plasma membrane that untimely open, is a continuous threat. Figure 1 illustrates this primordial Ca^2+^ gradient and the additional intracellular Ca^2+^ gradients that can be generated over the membranes of cell organelles in which various types of enzymes are anchored: Endoplasmic Reticulum (RER and SER), Golgi, mitochondria and (occasionally) even the nucleus. In addition, there is also the risk that [Ca^2+^]i will rise for too long above a toxic level when Ca^2+^ is released from internal stores, e.g., by the common mechanism of Ca^2+^-induced Ca^2+^-release. As such, excess Ca^2+^ has to be removed as quickly as possible. The key tool for this purpose is the removal of Ca^2+^ by Ca^2+^-pumps located in the plasma membrane and in internal membranes lining the ER (e.g., the SERCA pump) and mitochondria, organelles that can act as temporary Ca^2+^ -storage sites. A second mechanism is to restrict as much as possible the passive entry of Ca^2+^ through all Ca^2+^-channels. For figures, see De Loof et al. (2014) and De Loof (2017). In this paper, the role of farnesol-like (FLS) endogenous sesquiterpenoids, in particular the juvenile hormones of insects (Qu et al., 2018), as restrictors of Ca^2+^-entry will be emphasized.

## Identifying a Second Problem: Not Only *Endocrine* JH Exists, but *Exocrine* JH as Well

**FIGURE 2:**
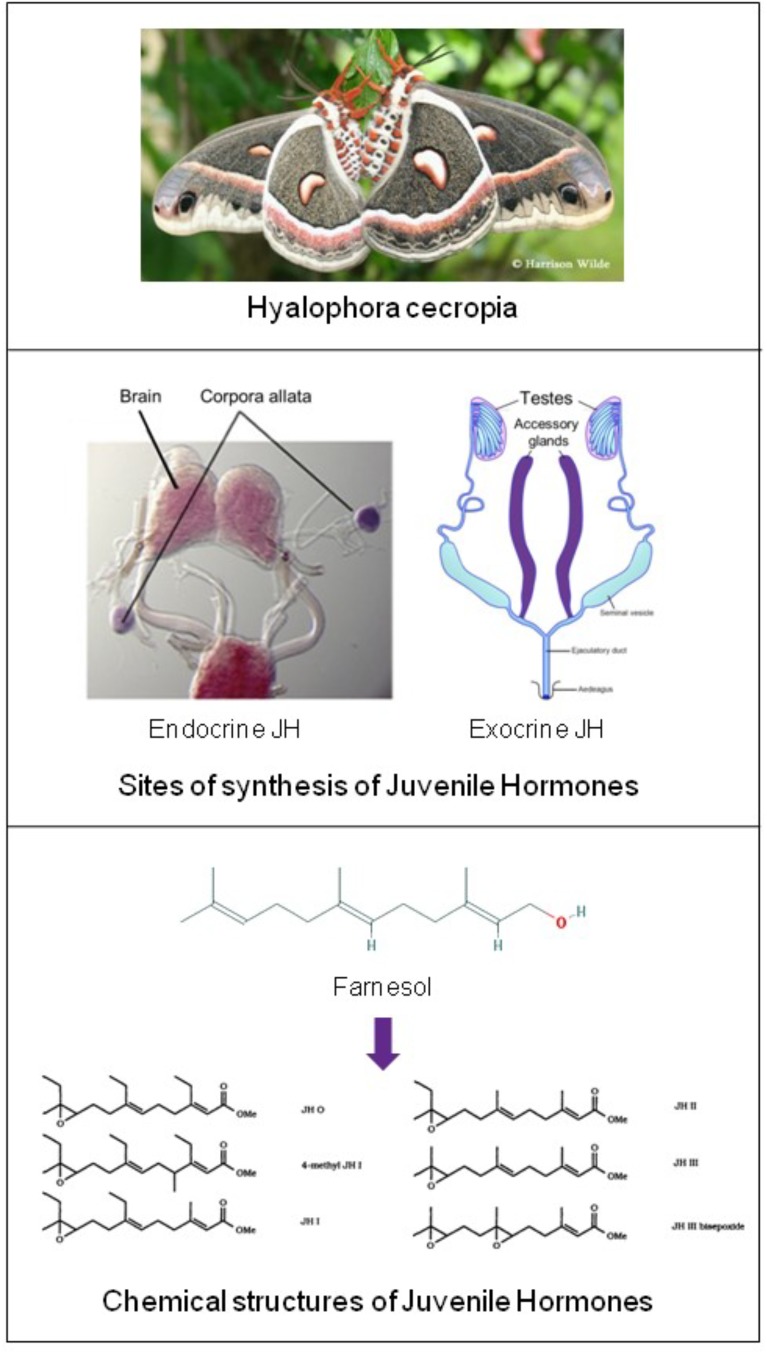
Top: *Hyalophora cecropia*: in extracts from abdomens of adult males of this lepidopteran species Juvenile Hormone-I was chemically identified for the first time. Two sites of JH synthesis are known: the major site are the Corpora Allata (CA) which are present in all insects. CA do not accumulate JHs. CA-JH is endocrine JH. *Cecropia* Male Accessory (colleterial) Glands (MAGs) also synthesize JHs (Paroulek and Sláma, 2014), and they accumulate high amounts of JHs, in particular JH-I. Hitherto, JH synthesis by MAGs (= exocrine JH) has only been demonstrated in a few species (*Hyalophora cecropia* and *Aedes aegypti*). Top: Picture of *H. cecropia*: From Google images: Butterflies and Moths of North America. Collecting and sharing data about Lepidoptera (Photographer Harrison Wilde). Middle: Sites of synthesis of JH (in purple) Corpora allata of the silk moth *Bombyx* (From Google images: authors Daimon et al., 2012); Male accessory glands: image slightly modified after Google images: Cronodon.com – Insect Reproduction- (BotRejectsInc). Bottom: Linear chemical structures of farnesol [*Trans,trans*-Farnesol or (E,E)-Farnesol] (From PubChem) and of Juvenile Hormones (JHs): Slightly modified after Bede and Tobe (2000). Their horseshoe 3D configuration is shown in Figure 7. Thanks to all authors of the original figures. Copyright permission not required.

In 1947 Carroll Williams in Harvard discovered the first materials with juvenile hormone (JH) activity. He found high activity in lipid extracts from abdomens of adult male, but not of female, *Hyalophora cecropia* silkworms (Figure 2) (Williams, 1956; Paroulek and Sláma, 2014). At that time the only known site of synthesis of juvenile hormone were the Corpora Allata.

(CA: tiny glands located in the head: Figure 2). Hence, Williams and others assumed that the JH-active material was synthesized in the CA with subsequent transport to, and accumulation in the abdomen, more specifically in the male accessory glands (MAGs) (Williams, 1956; Shirk et al., 1976). Thus in this view, MAGs are only a repository for JH. The active factor turned out to be 10,11-epoxyhomofarnesoate ester (IUPAC name: methyl (2E,6E)-7-ethyl-9[(2R,3S)-3-ethyl-3-methyloxiran-2-yl]-3-methylnona-2,6-dienoate) that was named JH-I (Röller and Dahm, 1968). Its chemical identification and the elucidation of its synthesis from farnesol as precursor in the mevalonate pathway were true landmarks in endocrinology. Because of its activity in bioassays designed to detect JH activity, the *Cecropia* MAG-factor was eventually named “Juvenile Hormone I” (JH-I = the first discovered JH). This is a common practice: hormones are usually named after their first detected biological activity (Peferoen and De Loof, 1980). Later other JH-isoforms were found, as well other compounds that were active in JH bioassays. Some were endogenous in insects/arthropods or even in some plants (Bede et al., 2001), but the great majority (some 4000 were tested) were synthetics (Sláma, 2013).

However, since its initial discovery, doubts were raised as to whether MAG-JH met all the necessary requirements in order to be classified as a “true” hormone. Indeed endocrinologists wondered why huge amounts of a factor with hormonal activity accumulated in a gland that was not known as being part of the insect’s known *endocrine* system, but as an *exocrine* gland that secretes a variety of compounds into its own lumen, but not into the haemolymph (which would be the case if MAG-JH would act as a true hormone). The assumption that no endocrine role is attributed to the MAGs of animals in general, the human prostate inclusive, seems to be the rule. This view did not change much since its original formulation by Leopold (1976). Another cause of doubt was that the physiology of *Cecropia* seemed to be exceptional. In corast to most insect species in which MAGs start developing to full maturity much later, namely after adult eclosion, the MAGS of *H. cecropia* develop precociously during metamorphosis, enabling males to mate very soon after adult eclosion. Yet, in the mosquitoes *Aedes aegypti, Culex nigripalpus, Anopheles rangeli, and Anopheles trinkae* in which JH synthesis in male accessory glands has also been experimentally documented (Borovsky et al., 1994), the MAGs develop later than in *H. cecropia*.

Twenty years after Borovsky’s experimental data, Paroulek and Sláma (2014) presented a plausible answer to the exocrine JH-MAG question. They found that *Cecropia* MAGs synthesize JH by themselves, in the same way as the CA do in juveniles and in reproducing adult insects. They also confirmed - what had been assumed to be the case for decades - that *Cecropia* MAGs do not secrete JH into the haemolymph like the CA do, but that they transfer a substantial amount of their JH content during copulation into the female. This was deduced from the fact that the JH content in MAGs was much higher before than after mating. Thus the MAG-synthesized JH-I, the farnesol ester methyl 10,11-epoxyfarnesoate, is in fact an *exocrine secretion product but not a hormone* that erroneously was named Juvenile “*Hormone,”* while it had no hormonal function at all in MAGs. It follows that JHs must have another, non-hormonal function inside the reproductive system, and not necessarily in males only. Paroulek and Sláma (2014) made some suggestions that will be dealt with later (§8.4). In contrast, JH-I has the same chemical structure as MAG-JH, but is synthesized by the CA (CA-JH), and is actually released into the haemolymph, and thus acts as a true hormone. Thus the JH (JH-I) from the CA is *an endocrine secretion product, thus a hormone*. For completeness: the related sesquiterpenoids JH-II and JH-III were also found in the MAGs of *Cecropia* silkworms. They occur in substantially lower amounts, namely twelve times less JH-II than JH-I. The most widespread JH in insect species, JH-III, only occurs in very low amounts, or even under detectable analytical limits (Paroulek and Sláma, 2014).

In FlyBase that documents the genome of *Drosophila melanogaster*, data about the expression profile of juvenile hormone acid methyltransferase (Jhamt) are listed. This enzyme catalyzes (2E,6E)farnesoate/juvenile hormone (JH) and JH bisepoxide in the corpora allata. The highest expression of the coding gene as assessed by microarray is in the head, in particular in the brain. The second highest expression is found in the male accessory glands. In addition there is also some expression in the crop, midgut, hindgut, salivary glands, and in the testis. In females, the highest expression is situated in mated spermatheca, while values for virgin spermathecal are lower. However, when assessed by RNA-Seq, the highest expression is found in the male accessory glands of 4 days mated males, which is 4 times higher than Jhamt expression in the testis and 20-fold that of the head, the CNS and the digestive system. No data are available on whether the mRNA is translated into the corresponding protein in all these tissues.

## Questions and Hypotheses Arising From the Endo-Exocrine Dichotomy

For various reasons, but especially because “exocrine JH” is an exotic (and contradicting itself!) concept in insect endocrinology, several questions beg for an answer.

**FIGURE 3:**
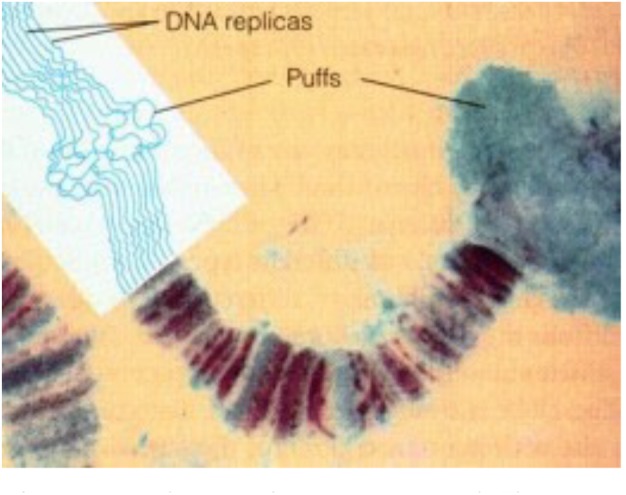
Polytene chromosome and Chromosome Puffs, also named Balbiani rings. During transcription DNA strands in bands can uncoil, or puff. Thus bands indicate the presence of (clusters of) genes, while interband regions are devoid of genes. Chromatin remodeling is part of the (in)activation of gene (clusters). From Google images (Biology exams 4U: author not mentioned). Copyright permission not required.

• CA-JH (endocrine) and MAG-JH (exocrine) are chemically identical, but do they also act in the same way, or alternatively do they act complementarily, or differently? For instance: Is it possible that the high concentration of JHs in the MAG-secretion of *H. cecropia* contributes to optimizing the *sperm’s fitness*?
• Sperm cells represent the essence of the male reproductive system. In case of internal fertilization, do the secretions of the MAGs/prostate help to ensure their optimal transport and survival during the transfer from the male to the eggs inside the female? For sperm cell-types having a flagellum: how does it keep undulating? Is Ca^2+^ instrumental to such activity as in case of ciliated cells in general (Delling et al., 2013; Pala et al., 2017)? Because the extracellular Ca^2+^-concentration is invariably higher than the cytoplasmic one, sperm cells face the danger of Ca^2+^-induced apoptosis: how do they prevent it?
• Animals are thought to descend from an ancient unicellular choanoflagellate (Cavalier-Smith, 2017) in which both voltage-gated Ca^2+^channels (Moran and Zakon, 2014) and the mevalonate biosynthetic pathway (Cheong et al., 2015) that yields farnesol were already operational. The typical cellular architecture of sperm cells, in general consisting of a head and an undulating flagellum, is evolutionarily ancient. It even dates back prior to the emergence of the proto-choanoflagellate. Thus, comparing animal sperm cells with choanoflagellates is not too far sought. All Opisthokonta, formerly also called Choanozoa, are organisms having flagellated cells (like sperm cells). The flagellum at their rear end (hence the name “Opisthokonta”) is formed from centrioles, which have a 9+2 microtubule-based configuration. Since it is now clear that some protists, sponges, all animals and Fungi form a monophyletic group, can part of the mode of action of both MAG- and CA-JH, be understood from the knowledge about the cell physiology of the flagella-cilia organelles, in particular with respect to their Ca^2+^- signaling (Cai, 2008; Delling et al., 2013)?
• Farnesol, the precursor of all juvenile hormones (JHs) and itself a compound with moderate JH activity (Wigglesworth, 1969), has been identified already two decades ago (in some mammalian cell types), as an endogenous inhibitor of some type of voltage-gated Ca^2+^ channels that act as their *plasma membrane receptor* (as already cited). *This raised as yet unanswered questions as to the cell physiologic archaeology and ancient role of the tandem voltage gated Ca^2+^-channel-farnesol/FLS.* Unicellular organisms do not have hormones by definition. Does the presence of JHs in *Cecropia* MAGs still reflect the original function of farnesol in ancestral flagellate cells? Are some of the known effects and mode of action of farnesol/FLS *as a hormone(s)* also derived from the mode of action of the tandem?
• Is farnesol an overlooked hormone and/or inbrome in humans and other vertebrates? The main problem in proving with certainty that farnesol/FLS might act as a hormone or as an “inbrome” (De Loof et al., 2015a) in vertebrates/mammals is that here *no situation can be created in which farnesol/FLS is completely absent from the whole body*. Such situation of total absence of farnesol and its esters occurs naturally at the onset of metamorphosis in the beginning of the last larval instar of all holometabolous insect species (Peferoen and De Loof, 1980). Hence insects are good experimental models in this respect.
• The most important issue is: Do JHs, known to be highly *hydrophobic* and to literally “stick to everything,” stainless steel, glass, plastics, lipid membranes and a variety of proteins (Prestwich et al., 1996), only activate transcription factors and selected genes *if they end up at a very specific location inside the nucleus*? In addition, how can a hydrophobic molecule end up in the nucleus without a transport system or carrier?
• With respect to nuclear receptors for JHs (more information in §8.2): If the nuclear receptor named Met/Tai is a transcription factor complex of which the conformation and activity, along with that of chromatin (Lai et al., 2009), is sensitive to changes in the intranuclear [Ca^2+^], then the migration of JHs into the nucleus may not even be required. Or might it be possible that the changes in [Ca^2+^]i, namely the inhibition of the influx of Ca^2+^, brought about by the binding of farnesol/FLS to their membrane receptor (in mammals this is a Ca^2+^ channel-type) and, in addition, to changes in the intracellular phosphorylation pathways (Jindra et al., 2015b) suffice for causing the 3D changes of chromatin and in the conformation of Met/Tai? *In other words, could it be that the transcription factor complex Met/Tai is Ca^2+^ sensitive, and that it needs the help of Ca^2+^-calmodulin, an element of the chromatin remodeling complex, so that different sets of genes are transcribed/inhibited at low [Ca^2+^]i (= larval stages) versus at high [Ca^2+^]i (= metamorphosis and reproductive state)*? This mode of action is (partially?) compatible with the fact that the very fast effects of binding of JHs to their membrane receptor(s) precede the much slower effects via transcription. The younger generation of insect endocrinologists is probably not aware of the experimental work of Lezzi and Kroeger some 50 years ago (= the old Lezzi-Kroeger hypothesis: for refs see De Loof et al., 2014), who demonstrated that some of the so called “ecdysone- or JH-specific” puffs in the polytene chromosomes (Figure 3) of some dipteran insects, which were thought to visualize changes in gene expression, could be induced *in the absence of any hormone*, by simply changing the inorganic ion composition of the medium in which the salivary glands were incubated. Thus, the presence of the hormone inside the nucleus is not an absolute prerequisite for hormone dependent activation of selected genes.

## Farnesol and Farnesol-Like Substances(FLS)

### Naming, Chemistry and Biosynthesis in the Mevalonate Pathway

**FIGURE 4:**
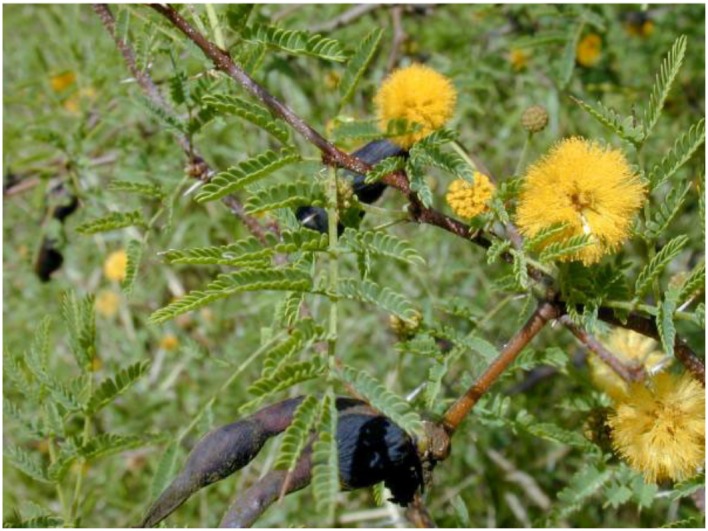
*Vachellia farnesiana*, or the Farnese acacia tree, the plant after which “farnesol” was originally named (Wikipedia: Farnesol). From Wikimedia Commons: File: Acaciafarnesiana1web.jpg. Is in the Public domain, no copyright permission required. With thanks.

**FIGURE 5:**
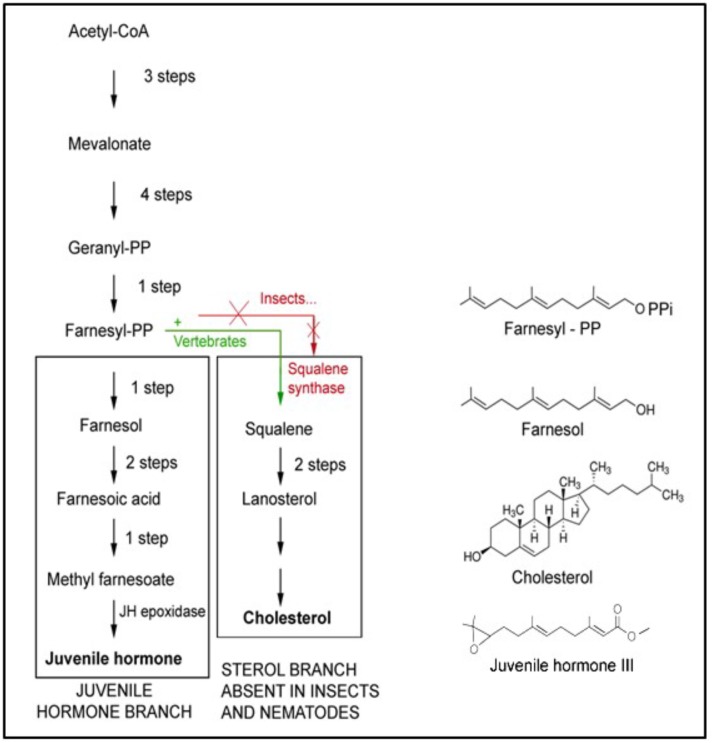
The mevalonate biosynthetic pathway. It operates in all eukaryotes, thus mammals and humans inclusive, but with some “branch-specific “variability that is mainly linked to the presence or absence of the gene coding for the enzyme squalene synthase that is needed for the synthesis of squalene. This is the precursor of cholesterol, with its many functions, e.g., in the synthesis of sterols and “vertebrate-type steroid hormones”. Insects, nematodes and some other species cannot synthesize squalene. Hence, cholesterol is a vitamin for them. They 1215 1216 convert farnesol into esters (Figure 2) whose juvenile hormone activity (in bioassays) is much 1217 higher than the mild one of farnesol itself. Adapted from De Loof et al., 2014, 2015a). Copyright permission not required.

Farnesol is a 15-carbon acyclic sesquiterpene alcohol that was originally extracted from the Farnese acacia tree, *Vachellia farnesiana*, around 1900 (Figure 4). It is a colorless liquid, hydrophobic, and thus immiscible in water. It is best known from its use in the perfume industry. It is present in essential oils originating from a variety of plant species. It is used to emphasize the odors of sweet floral perfumes as an enhancer of perfume scent (Wikipedia: 359 Farnesol). Later, after having been identified in plant extracts, farnesol was found in all eukaryotes in which it was searched for. This is not surprising, because it is a side product in the ubiquitous biosynthetic pathway of mevalonate-farnesylpyrophosphate pathway (Figure 5) that also yields cholesterol and steroids in vertebrates but not in arthropods and nematodes. In insects this pathway yields farnesylpyrophosphate, farnesol and juvenile hormone(s), which are esters of farnesol (Qu et al., 2018). The dichotomy in the mevalonate biosynthetic pathway existing between insects and vertebrates requires some explanation. The key difference resides in their ability to synthesize cholesterol by themselves, or not.

Plants and Ecdysozoa (= nematodes and arthropods) cannot synthesize cholesterol, because they lack the gene coding for squalene synthase. Vertebrates have this gene, hence they can synthesize cholesterol and use it as an intermediate in the synthesis of vertebrate steroids, e.g., of the sex steroids testosterone and estradiol. Insects also have sex steroids, but they belong to the family of the ecdysteroids (De Loof and Huybrechts, 1998; De Loof, 2015). These also occur in plants, but here their role is poorly understood. The cited dichotomy raises intriguing questions. Did ancestral insects lose the squalene synthase gene in evolution, or did the acquisition of this gene in chordates/vertebrates represent a late development in evolution? We favor the view that the situation in plants and in insects, which are evolutionarily older than chordates, might have been the original one. This view implies that the synthesis of cholesterol cannot have been the main issue in the ancient mevalonate pathway. Because not all eukaryotes convert farnesyl-pyrophosphate (FPP) into farnesol esters with potent juvenile hormone activity, the synthesis of JHs cannot have been of prime importance either. Thus the probable explanation is that *farnesol itself represents the very heart of the mevalonate pathway*. Hitherto, it is largely undervalued that it is one of the key players in controlling Ca^2+^-homeostasis (Roullet et al., 1999).

### 3D Structure: Horseshoe Shape, Highly Flexible (Rotatable Bond Count of 7)

The 3D structure is important for understanding how farnesol binds to its receptors, and how it exerts its function(s) at the level of the plasma membrane. A parameter instrumental to the functioning of a ligand that is seldom mentioned is the “rotatable bond count” of the ligand (7 for farnesol and 10 for JH I: PubChem). The definition given in the “Molinspiration” website reads: “Rotatable bond is defined as any single non-ring bond, bounded to nonterminal heavy (i.e., non-hydrogen) atom. Amide C-N bonds are not considered because of their high rotational energy barrier.” This simple topological parameter is a measure of molecular flexibility.

**FIGURE 6:**
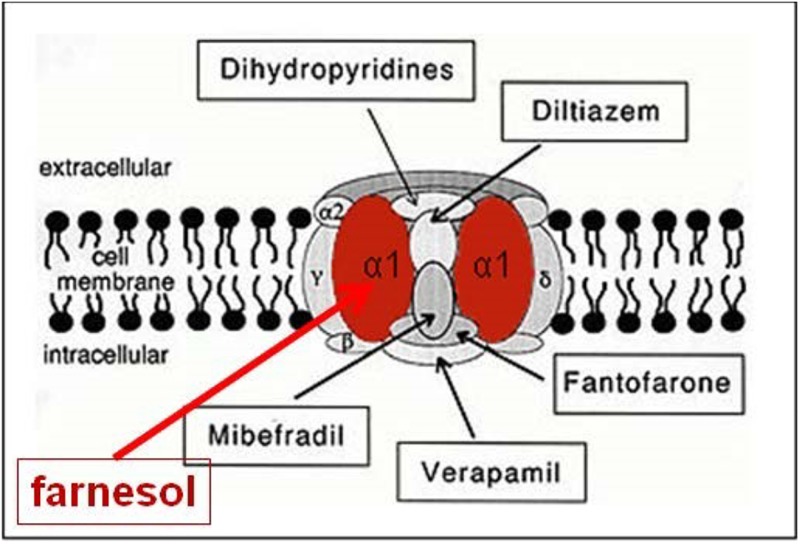
Farnesol is an inhibitor of some types of voltage-gated Ca^2+^ channels. Roullet et al., 1999, and Luft et al., 1999 demonstrated for the first time that farnesol binds to the _α_1 subunit, which is the pore forming unit of voltage-gated Ca^2+^ channels. Ligands for the other subunits are well documented (Figure modified after Wikipedia: Calcium channel). Copyright permission not required.

**FIGURE 7:**
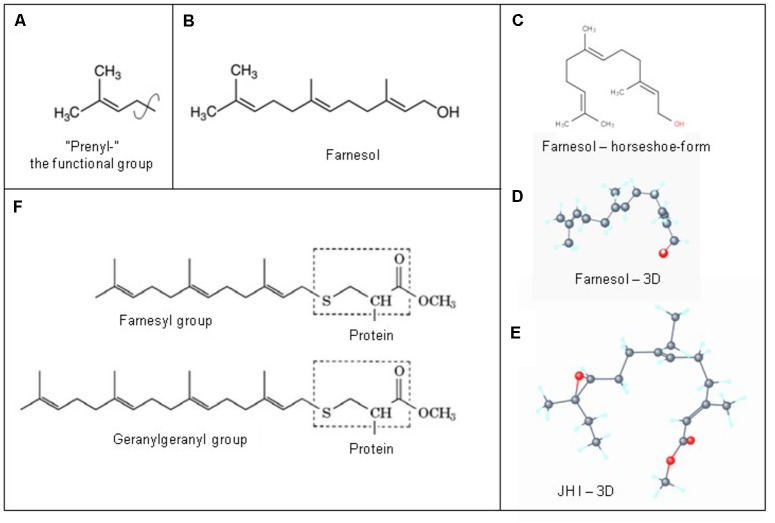
Prenylation. **(A)** The chemical structure of a “prenyl”-group and of farnesol. **(B)** In textbooks farnesol is usually depicted in its linear 2D configuration. **(C)** Its 3D conformation is horseshoe-shaped. **(D,E)**: the 3D configuration showing all atoms of all-trans-farnesol and of Juvenile Hormone I (JH I) (according to PubChem). **(F)** Protein prenylation is the covalent addition of a farnesyl- or geranyl-geranyl moiety to the C terminus of specific proteins, e.g., _α_ or _γ_ G protein. Adapted from Wikipedia: Prenylation, from Wikipedia: Farnesol and from PubChem: Farnesol and Juvenile Hormone I. With thanks. Copyright permission not required.

When the farnesol receptor, namely the pore forming α1 unit of a voltage-gated Ca^2+^ channel (Figure 6), was described by Roullet et al. (1999) and Luft et al. (1999) (§5.3), this parameter was not mentioned. De Loof (2017) pointed to its possible importance upon observing that farnesol, JHs and other compounds with juvenile hormone activity, all have a very similar horseshoe shape (Figure 7). In addition single and double bonds alternate in a similar pattern in farnesol and FLS. These observations prompted us to suggest that farnesol and its JH-esters might possibly function as flexible “molecular valves” controlling the passage of selected solutes through transmembrane helix bundle proteins. After an activity cycle of an ion pump or channel, the pore forming loops (usually three) is be brought back into a resting, tightly closed position to minimize passive leakage of inorganic ions or other solutes. This possible function matches well the data of Roullet et al. (1999) and Luft et al. (1999) that farnesol inhibits some types of Ca^2+^-channels by keeping them in the closed position.

This mode of action may not be restricted to the Roullet-Luft type channel. Farnesol is a sesquiterpenoid like the potent SERCA-Ca^2+^ blocker thapsigargin. Both the presence of thapsigargin and absence of JH/FLS (JHs) *induce Ca^2+^-induced apoptosis*, among others required for metamorphosis in holometabolous insects. Thus the SERCA pump, in its uninhibited, normal state, may need farnesol/FLS to keep the intraluminal [Ca^2+^] high in the endoplasmic reticulum (SER, RER). Furthermore, given the high degree of molecular conservation among various types of Ca^2+^-channels, it may be possible that the ryanodine-sensitive Ca^2+^-channel which resides in membranes of the endoplasmic reticulum, may also need farnesol for keeping it in the closed state. Thus, a link of controlling the influx of Ca^2+^ concurrently at the level of the plasma membrane and the intracellular membrane systems is mandatory, otherwise the entire Ca^2+^-homeostasis would collapse. Several questions remain to be explored, e.g., how does the horseshoe-shape of farnesol, if it enters the nucleus, play a role in effects on transcription?

### The Identity of the Membrane Receptor(s) of Farnesol in a Rodent Vascular System

Both Luft et al. (1999) and Roullet et al. (1999) using patch clamp techniques on transfected HEK -, rat aortic A7r5-, and Chines hamster ovary cells expressing several types of Ca^2+^ channels (neuronal voltage-dependent- and smooth muscle Ca^2+^ channel alpha 1C subunits), reported that farnesol acts as an inhibitor of a voltage-gated Ca^2+^-channel(s), more specifically as a selective, high affinity inhibitor of N-type Ca^2+^ channels. Roullet et al. (1999) reported concentrations of 100–800 pmol/g (wet weight) of farnesol in the brain of rodents and humans. They raised the possibility that endogenous farnesol and the mevalonate pathway are implicated in neurotransmitter release through regulation of presynaptic voltage-gated channels. Luft et al. (1999) concluded that farnesol may represent an endogenous smooth muscle L-type Ca^2+^ channel antagonist that targets the alpha 1C subunit, which represents the heart of the pore forming complex. The evolutionary roots of the four subunits of voltage-gated calcium channels have been well studied by Moran and Zakon (2014).

There is no reason to assume that the way farnesol acts in mammalian cells, would be much different in insects. Indeed, both the mevalonate pathway and voltage-gated Ca^2+^ channels are evolutionarily ancient: they date from before the split between plants and Opisthokonts and before animals with their excitable muscle- and nerve cells appeared on the scene (Moran and Zakon, 2014). This suggests that the tandem voltage-gated Ca^2+^ channels with farnesol may have been shaped to near functional perfection already hundreds of millions years ago, *with the locomotor and feeding complex of the eukaryotic flagellum/cilium (e.g., in Choanoflagellates) as one of its main targets*.

Noteworthy is that no membrane receptor for farnesol esters known as JHs has so far been identified by either classical biochemical- or by electrophysiological methods. However, a plausible extrapolation about its identity can be made from the electrophysiological 490 characterization of the membrane receptor of farnesol, the precursor of all JHs that itself has 491 moderate JH activity (Wigglesworth, 1969).

## Farnesol/FLS May Also Act From the Intracellular Cytoplasmic Side as a Flip-Flopping” Farnesyl- Valve That is Able to Restrict the Untimely Influx of Excess Ca^2+^

Farnesol as a hormone starts acting at the extracellular side of cells, when blood contacts the plasma membrane. Yet, there is another equally important possible mechanism of action, namely at the border between the cytoplasm at the inner side of the plasma membrane with its numerous embedded helix bundle transmembrane proteins, in particular the G Protein-Coupled Receptors (GPCRs) and their associated G-proteins. Prenylation, which is also named as “lipidation,” is the likely mechanism involved. Prenylation is the covalent addition of hydrophobic molecules to a protein or chemical compound (Zhang and Casey, 1996) (Figure 7). Protein prenylation involves the transfer of either a farnesyl or a geranyl-geranyl moiety to a C-terminal cysteine(s) of a target protein. Examples of well documented targets of prenylation are the α and γ subunits of G-proteins of heterotrimeric G protein complexes, *Ras*, which play a central role in the development of cancer, and the nuclear lamin having a role in Hutchinson-Gilford progeria in humans.

The usual functions that are ascribed to prenylation are mediating protein–protein interactions and protein–membrane interactions (Wikipedia: Prenylation). In particular the finding that farnesol is an endogenous inhibitor of some types of voltage-gated Ca^2+^-channels triggered the hypothesis that farnesylation of G-proteins may, perhaps serve a similar function, but acting at the cytoplasmic side of the membrane. A re-analysis of the existing literature yielded the proposal that a farnesyl/prenyl group covalently attached to a G protein (α or γ), upon being inserted in the intramolecular microchannel that is present in any GPCR, may act as a possible “flip-flopping” *flexible molecular valve that can restrict the untimely influx of excess Ca^2+^* through the microchannel inside the GPCRs. This supposed mode of action can be compared with screwing a moderately flexile cork into a wine bottle.

This has to be seen in a cell-physiologic archaeological context: the majority of GPCRs are thought to be the molecular progeny of microbial rhodopsins with a function in transmolecular H^+^ transport, and a covalently attached farnesyl-group likely functions as the functional substitute for microbial rhodopsin’s retinal. Like flip-flopping retinal acts as a valve for restricting H^+^ transmembrane transport in prokaryotic rhodopsins, so may a prenyl/farnesyl- do for restricting Ca^2+^ transmembrane transport of Ca^2+^.

The novel “flip-flop concept” that approaches the problem of ligand-associated allosteric changes in GPCR-signaling in an alternative way as compared to contemporary methods (e.g., Thal et al., 2018), will be described in more detail elsewhere.

## Nuclear Receptors for Farnesol, Its Esters (= JHs) and Metabolites

The fact that all-trans-farnesol is active in bioassays for juvenile hormone (Wigglesworth, 1969; De Loof et al., 2014) proves that one way or another farnesol has an effect on transcription of particular genes, e.g., genes coding for “pupal” cuticular at the moment that the epidermis is already synthesizing adult-type cuticular proteins during metamorphosis as evidenced by the *Galleria* bioassay for JH (De Loof et al., 2014). To our knowledge, a nuclear receptor for farnesol has not yet been identified in contrast to its membrane receptor, (voltage-gated Ca^2+^ channels:Luft et al., 1999; Roullet et al., 1999). The situation for JHs is just the opposite.

Here the current view is that the mode of action of JHs can be explained in full through the action of nuclear receptors. In the following sections we will outline that such a reductionist mode of action in which there is no role for membrane receptors is likely to be very incomplete.

### The Evolutionary Origin of “Juvenile Hormones”

All 6 known insect/arthropod juvenile hormones are simple esters of farnesol. Enzymes that can form such esters are not confined to insects/arthropods but also occur in some plants, e.g., the sedge *Cyperus iria* (Bede et al., 2001). This suggests that the ancient key function(s) of the JH esters was probably non-hormonal. In bioassays that detect JH bioactivity, JHs are orders of magnitude more active than all-trans farnesol, the most active farnesol isomer (Wigglesworth, 1969; Peferoen and De Loof, 1980). This may explain why JH synthesis can be restricted to a tiny gland in the body, namely the corpora allata, which are part of the retrocerebral complex. A second feature contributes to the drastic effect that JHs can exert on physiology, namely that the synthesis of JHs can be completely shut down at some moment in larval/juvenile development. This shut down, in combination with the clearance from the body of all circulating JH molecules is the primordial inducer of complete metamorphosis in all holometabolous insects. Several causes of the inactivation of the CA are known or have been suggested: secretion of allatostatins, inhibition of allatototropins, complete absence of short Neuropeptide F (sNPF) (Huybrechts et al., 2004; Caers et al., 2016). The situation in which all JH disappears from the body is ideal in order to uncover which physiological processes are inhibited by a high JH titre as present in young larvae. As already stated, in vertebrates, there is no phase in development in which the body stops producing farnesol/FLS. This is the major reason why some of farnesol’s functions remained hidden for so long (De Loof et al., 2015a).

### Classical View on JHs as Ligands for Nuclear Receptors

The search for nuclear receptors for the two key hormones controlling insect development in general and metamorphosis in particular, namely ecdysteroids and juvenile hormones, has been more successful than the one for their membrane receptors. The nuclear ecdysone receptor (EcR) forms a dimer with ultraspiracle (USP) (Devarakonda et al., 2003). The best documented nuclear JH receptors are Methoprene-tolerant (Met) with its binding partner Taiman (Tai) and Gce (Charles et al., 2011; Lozano et al., 2014; Jindra et al., 2015a,b; Kayukawa et al., 2017; Lenaerts et al., 2018; Li et al., 2018). Their exact mode of action in the nucleus is not yet fully understood (Jindra et al., 2015a). This receptor-type may be rather irrelevant for understanding the mode of action of exocrine JH on sperm cells (see later). In *Drosophila* the JH-resistance gene *Met* codes for a transcription factor that plays a critical role in insect metamorphosis. Also in *Drosophila* a paralogous gene to *Met*, namely *Germ cell-expressed* (*gce*) has been found. Its effects are partially redundant in transducing JH action (Abdou et al., 2011). The MET protein has the capacity to bind JH, which has been mapped to a particular ligand-binding domain, thus establishing this bHLHPAS protein as a novel type of an intracellular hormone receptor. MET has a receptor coactivator (RC, also known as FISC or Taiman). The JH/MET/Taiman complex binds to JH response elements present in the promotor regions of JH responsive genes including the Krüppel homolog I (*Kr-h1*). Both *met* and *gce* null mutants are fully viable, but the *met-gce* double mutant dies during the larval-pupal transition. Exogenous application of JH agonists rescued the JH-deficient animals but not the *met(27)gce(2.5k)* mutants (Abdou et al., 2011). Here it should be noted that it follows from the findings of Roullet et al. (1999) that farnesol, and by extension its esters (the JHs) are inhibitors of some types of voltage-gated Ca^2+^ channels, and that farnesyl- plays an important role in prenylation. Hence, exogenous application of a JH agonist *drastically changes the entire Ca^2+^-homeostasis system* and as such, it is not an appropriate rescue method for the cited null mutants.

### Can a Change in Intranuclear Ca^2+^ by Itself Play a Role in Transcription, Thus Without Needing the Presence of a Hormone in the Nucleus?

An unanswered key question whether Met/Tai is a Ca^2+^ -sensitive transcription factor complex that controls different sets of genes being transcribed at lower Ca^2+^ levels (= when the JH titre is high) versus at higher intranuclear Ca^2+^ concentrations (= e.g., when JH is absent or/and when the ecdysteroid titre peaks)? This differential activity happens in concert with conformational changes in chromatin which are also known to be Ca^2+^-dependent.

• In contemporary insect endocrinology, the focus is almost exclusively on nuclear receptors for explaining the mode of action of both JH and ecdysteroids. The nuclear ecdysone receptor (EcR) forms a dimer with ultraspiracle (USP) (Devarakonda et al., 2003), and Met/Taiman and Gce are thought to be the nuclear receptors for JHs. No role for Ca^2+^ signaling in the activation of these receptors was reported. However, both farnesol (Roullet et al., 1999) and probably its esters as well, and 20E first act at the level of the plasma membrane and antagonistically use Ca^2+^-signaling (Cai et al., 2014; Wang et al., 2015, 2016). Is it conceivable that changing cytoplasmic [Ca^2+^] by itself can stimulate or inhibit the transcriptional activity in the nucleus? Half a century ago, Markus Lezzi and Heinrich Kroeger argued that this might be the case (§5 and Figure 3). Their theory on a role for inorganic ions in control of gene expression is forgotten. Yet, the issue whether e.g., changing intracellular/intranuclear [Ca^2+^] can by itself change transcriptional activity is continued to be explored.

• Thiel et al. (2012) showed that elevated extracellular Ca^2+^ concentrations stimulate the G-protein coupled receptor calcium-sensing receptor. This results in the induction of the expression of biologically active early growth factor response protein 1 (Egr-1), a zinc finger transcription factor. Two other transcription factors, Elk-1 and AP-1, were also upregulated by stimulation of the calcium sensing receptor. The intracellular signaling pathway includes the protein kinases Raf and ERK. The authors stated that regulation of gene transcription is an integral part of calcium-sensing receptor induced signaling. Comparable results were reported by Backes et al. (2018), who found that stimulation of TRPV1 channels (which play a role in pain sensation and inflammatory thermal hyperalgesia) by capsicain or resiniferatoxin induced an influx of Ca^2+^ into the cells, and that this rise in [Ca^2+^]i is essential for activating transcription factor AP-1.

• Effects of [Ca^2+^] on chromatin remodeling are also important. Lai et al. (2009) reported that in mammals the Swi/Snf-like BAF chromatin remodeling complex by itself is not sufficient for the chromatin remodeling that makes transcription of TLR4 target genes possible. Indeed, calcium/calmodulin is also required. It binds the HMG domain of the BAF57 subunit within the BAF complex.

• An indirect argument in favor of a direct effect of Ca^2+^ on transcription was shown by Liu et al. (2009) who reported that JH counteracts the transcription factors MET and GCE to prevent caspase-dependent programmed cell death in *Drosophila*. When at the onset of metamorphosis the insect body is completely cleared from compounds with JH activity (farnesol and JHs), the inhibitory effect of these hormones on voltage-gated Ca^2+^ channels (= suggested from the results of Roullet et al., 1999) is lifted, with influx of Ca^2+^ into the cytoplasm of target cells (fat body e.g.) and Ca^2+^-induced cell death/apoptosis (Orrenius et al., 2003). One should keep in mind that in stages of development where JH is absent, the JH receptor concept has no meaning. If MET and GCE induce programmed cell death by upregulating Dronc and Drice (Liu et al., 2009) in animals deficient in JH activity, the plausible explanation may be that it is the concentration in the nucleus of Ca^2+^ that is instrumental to the activity of the two transcription factors. When the JH titre is high, and as a consequence when [Ca^2+^] is low, they cannot activate the transcription of Dronc and Drice, which are both caspases genes that are crucial for programmed cell death. In contrast, when the JH titre drops to zero, both the titre of ecdysteroids and [Ca^2+^] rise thereby upregulating transcription of Dronc and Drice. The discovery of a membrane receptor (family) for ecdysteroids (named ErGPCR-2) that promotes the influx entry of Ca^2+^ into cells, activation of the phosphorylation signaling cascade etc. by Cai et al. (2014), Ren et al. (2014), Wang et al. (2015, 2016), and Liu et al. (2018) strengthens the view that JH signaling pathway also starts at the plasma membrane like the one for ecdysteroids. *This implies that that MET and GCE are likely Ca^2+^-dependent transcription factors, either because they are themselves Ca^2+^-sensitive, or/and because the accompanying chromatin remodeling complex requires calcium/calmodulin* (Lai et al., 2009).

• The fact that the nucleus can be temporarily and quantitatively be compartmentalized for Ca^2+^ (Meyer et al., 1995; Badminton et al., 1998, and others) suggests that such feature has a distinct role. The nucleus and nuclear envelope contain proteins instrumental to both regulating and responding to changes in [Ca^2+^]n. Nuclear pores enable sustained perinuclear calcium oscillations (Martins et al., 2016). Badminton et al. (1998) demonstrated that different stimuli induce changes in [Ca^2+^] in the nucleus and [Ca^2+^] in the cytoplasm that vary both temporally and in magnitude. The nucleus appeared to be shielded from increases in [Ca^2+^]c, either through a mechanism involving the nuclear envelope or by cytosolic buffering of localized increases in [Ca^2+^]. In addition, agonist stimulation resulted in an increase in [Ca^2+^]n. This is consistent with release from the perinuclear Ca^2+^ store. There is a stimulus-dependence between [Ca^2+^]n and [Ca^2+^]c suggesting differential regulation of [Ca^2+^]n.

### The Mode of Action of Insect Farnesol/FLS as an Inbrome: Non-genomic Effects

JHs and farnesol have an influence on lipid-, steroid-, protein- etc. biosynthesis. Part of the enzymes involved reside in intracellular membranes, in particular in the endoplasmic reticulum. Their activity partially depends on the Ca^2+^ concentration inside the lumen of the (S)ER. When JH concentrations are high, the intraluminal Ca^2+^ concentration is high, and Ca^2+^-sensitive enzymes involved are inhibited. When the Ca^2+^-gradient/concentration decreases, the inhibition is lifted. These are non-genomic effects, meaning that they do not require instant transcriptional activity in the nucleus (De Loof, 2017). Novel insights on the link between JH and Ca^2+^ homeostasis and on non-genomic effects of JH were gained during the re-examination of the role of JH, or better of its disappearance from the body, in inducing metamorphosis in holometabolous insects (De Loof et al., 2014, 2015b).

## Occurrence and Tentative Roles of Farnesol/FLS in the Reproductive System of Male Animals

### The Secretions of Accessory Glands Are Complex Mixtures

Accessory glands occur in both sexes. Their architecture varies from simple to very complex. Their secretion products can be very diverse. All male animals use accessory glands to facilitate the development, maturation, protection, storage and transport of sperm cells.

Compositional analysis of the luminal fluids collected from the epididymis (Zhou et al., 2018) and prostate (Hayward and Cunha, 2000; Veveris-Lowe et al., 2007; Wikipedia: The prostate) of a variety of vertebrate/mammalian species as well as from the MAGS of a variety of invertebrate species (Leopold, 1976; Happ, 1992; Gorskhov et al., 2015; Sharma et al., 2017) has revealed the complexity of the intraluminal milieu, with a diversity of inorganic ions, proteins, and small non-coding RNA transcripts etc. Most attention was given to MAG-proteins and peptides (e.g., the sex peptide of *Drosophila:* Denis et al., 2017). It is striking that in the literature the presence of farnesol-derivatives in MAGs is only sporadically mentioned, namely of JHs in the insects *H. cecropia* (Shirk et al., 1976), in *Aedes aegypti, Culex nigripalpus, Anopheles rangeli, and Anopheles trinkae* (Borovsky et al., 1994), and only indirectly in FlyBase of *Drosophila* (§3) without experimental results on possible functions. As mentioned before, De Loof et al., 2015a undertook an orienting analysis on the occurrence of farnesol in the prostate of mice. Farnesol could be identified qualitatively. No quantification was done. The medical literature on farnesol/prostate is limited, and mostly relates to some aspects of prostate cancer treatment (see later).

### Farnesol/FLS and JHS: Which Potential Role(s) in Male Accessory Glands?

The scarce knowledge to date about possible functions of farnesol/FLS in MAGS can be classified as “plausible guesses” that need to be experimentally validated. Paroulek and Sláma (2014) stated that the physiological meanings of the accumulation of sesquiterpenoid JH-I (and to a lesser extent of JH-II and JH-III as well) and of Vitamin E in the male ejaculate of *Hyalophora* are not completely clear. They suggested that selective advantages of sesquiterpenoids in insect reproduction can be envisaged in antioxidant properties, prolongation of sperm survival, fertilization, tanning of egg chorion, antimicrobial properties or prolonged survival of the fertilized eggs. The effect of farnesol on vascular smooth muscle in rodents (Luft et al., 1999) suggests that contractility of the muscles in the reproductive organs in both sexes should also be taken into account as a possible FLS target.

## Challenges to Sperm in the Genital Ducts of Both Males and Females

### The “Calcitox Concept” Also Applies to Sperm Cells

During their journey in the male genital system, and next upon arrival in female genital tract, the sperm cells must remain intact and fit enough for making contact with an egg cell. They must resist elimination by a variety of possible agents, high extracellular concentrations of Ca^2+^ inclusive. During this journey they retain their motility. This raises the question on how sperm cell motility is controlled. At first sight that the motility of the “tail” (flagellum or cilium or undulipodium) of a spermatozoon may look a rather simple, automated and long lasting undulating process. Yet, the opposite is true. The undulating movement requires energy and likely an oscillating influx/output of Ca^2^, not in the whole cytoplasm (Delling et al., 2013), but in a specific subcompartment. However, an influx of Ca^2+^ into the sperm’s cytoplasm *means the input of a toxic agent* (The Calcitox-concept: De Loof, 2017). How do sperm cells cope with an excess of toxic [Ca^2+^]i?

Like in all eukaryotic cells, the Ca^2+^ concentration in the cytoplasm of resting sperm cells must be kept very low, in the order of 100 nM. The extracellular Ca^2+^ concentration is likely in the order of millimolar, like in blood. Hence there is an enormous drive for Ca^2+^ in the sperm cell’s environment to passively enter the sperm’s cytoplasm through Ca^2+^-channels. The best strategy is to prevent Ca^2+^ from entering the sperm cell, or, if entry cannot be completely inhibited, excess Ca^2+^ must be removed to avoid cell death.

### The Complex Ca^2+^-Homeostasis System of Sperm Cells

**FIGURE 8:**
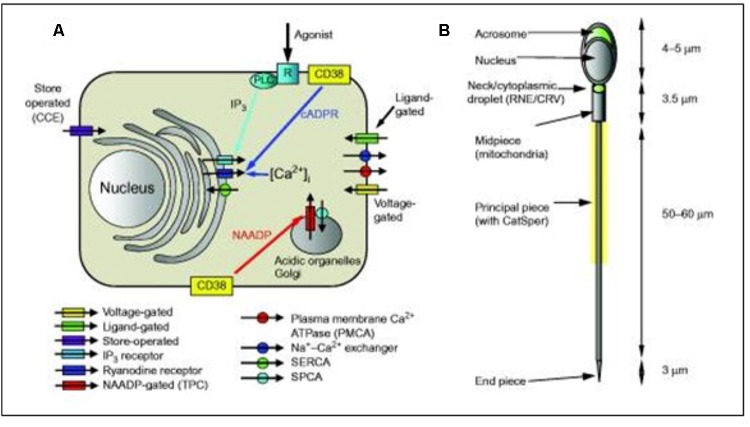
Simplified diagrammatic summary of [Ca^2+^]_i_ signaling toolkit in a somatic cell. Ion channels are shown as rectangles with arrow indicating normal direction of Ca^2^^+^ flow [yellow, voltage-gated; green, ligand-g **(A)** Ion channels are shown as rectangles with arrow indicating normal direction of Ca^2+^ flow (yellow, voltage-gated; green, ligand-gated; purple, store-operated; light blue, IP_3_ receptor; dark blue, ryanodine receptor; red, NAADP-gated]. Pumps are shown as circles with arrows indicating normal direction of Ca^2+^ movement (red, PMCA; blue, Na^+^–Ca^2+^ exchanger; green, SERCA; blue, SPCA). Activation of IP3 receptors by membrane receptor activation and phospholipase C is shown in light blue. Generation of cADPR and NAADP by CD38 and possibly other enzymes (leading to mobilization of Ca^2+^ from intracellular stores) is shown by yellow boxes. **(B)** Structure of human sperm showing positions of CatSper channels (yellow shading around anterior flagellum) and Ca^2+^ stores in the acrosome and at the sperm neck (redundant nuclear envelope and calreticulin-containing vesicles) (shown in green). From Correia J, Michelangeli F, Publicover S., 2015 Regulation and roles of Ca^2+^ stores in human sperm. Reproduction 150, R65-R76. With thanks for the copyright permission from both the authors and the publisher. Creative Commons Licensed.

Despite the very small size and apparent structural simplicity of spermatozoa, evidence is accumulating that they possess sophisticated mechanisms for regulating their cytoplasmic Ca^2+^ concentration and generation of complex Ca^2+^ signals. Correia et al. (2015) summarized the complexity of the sperm’s Ca^2+^-signaling ’toolkit’ (Figure 8). In particular, data accumulated over the last few years showed that spermatozoa possess one (and probably two) Ca^2+^ storage sites as well as a range of plasma membrane pumps and channels. Selective regulation of the various components of the toolkit by agonists probably enables spermatozoa to generate localized Ca^2+^ signals despite their very small cytoplasmic volume, permitting the discrete and selective activation of cell functions. In spermatozoa, several key functions, including acrosome reaction and motility, are regulated by the cytoplasmic Ca^2+^ concentration. According to Delling et al. (2013) the ionic conditions, permeability of the primary cilia membrane, and effectiveness of the diffusion barriers between the cilia and cell body are complex. Flagella/cilia are a unique calcium compartment regulated by a heteromeric TRP channel, PKD1L1–PKD2L1, in mice and humans. In contrast to the hypothesis that polycystin (PKD) channels initiate changes in ciliary calcium that are conducted into the cytoplasm, they showed that changes in ciliary calcium concentration occur without substantially altering global cytoplasmic calcium. PKD1L1–PKD2L1 acts as a ciliary calcium channel controlling ciliary Ca^2+^ concentration.

### Ca^2+^-Homeostasis of “Modern” Sperm Cells Looks Very Similar to That of “Ancient” Choanoflagellates

**FIGURE 9:**
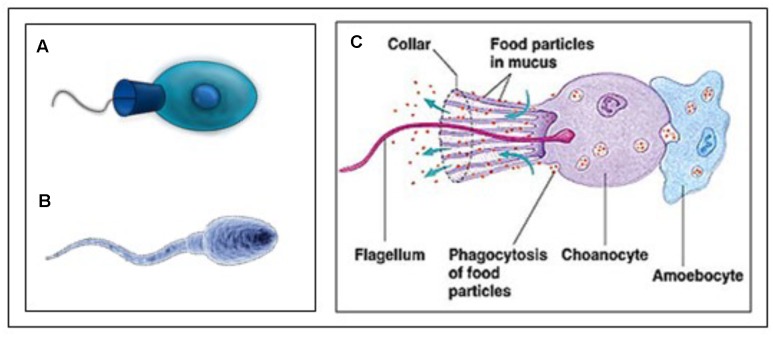
**(A)** Animal Choanoflagellate organism (Urutseg as original author (2009), licensed Under Creative Commons Attribution) and **(B)** Human spermatozoon: Google images, Kissping. Publicly available. **(C)** More detailed representation of an animal Choanoflagellate. From Google images and The wonderful World of Animalia, http://www.bio.miami.edu/dana/160/160S13_13print.html. Copyright permission not required. With thanks.

The reasoning underlying the comparison sperm cell – choanoflagellate (Figure 9) is: If we want to better understand the role of MAG-JH, we should ask which evolutionarily ancient functions could be stimulated by the presence of JH (or farnesol) or inhibited by its absence. Farnesol is very ancient in evolution. The type of membrane receptor inhibited by farnesol (and its esters), namely a voltage-gated Ca^2+^ channel type (among other candidates) is also evolutionarily ancient. It goes back at least as far as the Opisthokonta (or Choanozoa), unicellular organisms with a flagellum, which preceded the animals. Upon searching the literature, we found experimental data on the complexity of the Ca^2+^-homeostasis system in a choanoflagellate.

Cai (2008) described the presence of an extensive Ca^2+^ signaling toolkit in the unicellular choanoflagellate *Monosiga brevicollis* as follows. “Choanoflagellates possess homologs of various types of animal plasma membrane Ca^2+^ channels including the store-operated channel, ligand-operated channels, voltage-operated channels, second messenger-operated channels, and 5 out of 6 animal transient receptor potential channel families. Choanoflagellates also contain homologs of inositol 1,4,5-trisphosphate receptors. Furthermore, choanoflagellates master a complete set of Ca^2+^ removal systems including plasma membrane and sarco/endoplasmic reticulum Ca^2+^ ATPases and homologs of 3 animal cation/Ca^2+^ exchanger families.” Therefore, a complex Ca^2+^ signaling ‘toolkit’ might have evolved before the emergence of multicellular animals.

## Medical-Pharmacological Aspects

### Male Fertility. Farnesol and Human Prostate Cancer

It is estimated that infertility in humans is predominantly due to deficiencies situated in males. One in 15 men is sub-fertile and this frequency is increasing (Conner et al., 2007). Many causes for these deficiencies are known. Treatment is almost non-existent. To our knowledge, a role for farnesol in fertility has not been reported in the medical literature.

**FIGURE 10:**
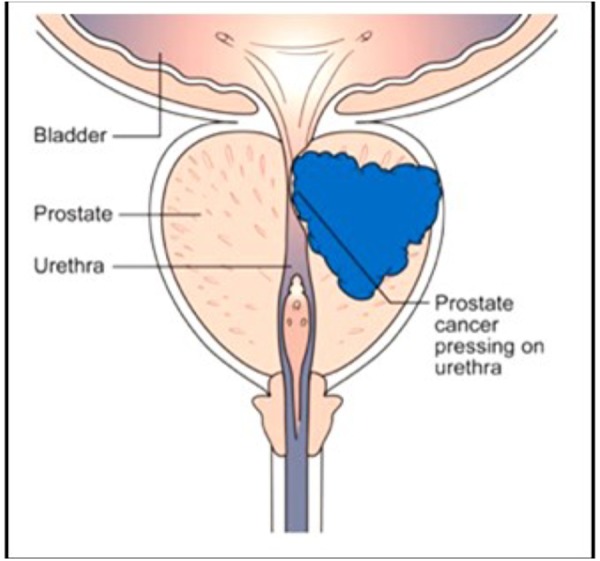
The human prostate: normal healthy gland (left side) and with a developing tumor (right side). Thanks to Cancer Research United Kingdom for the free use. No copyright permission required.

The human prostate gland is essential for male reproduction, but it is better known because of its association with prostate cancer (Figure 10) in aging man than because of its many functions in younger and older ones (Wikipedia: Prostate). De Loof et al. (2015a) investigated, with the same methods that were used to analyze mevalonate-biosynthesis in insects, whether farnesol is also be present in the prostate of mice. The outcome was positive (De Loof et al., 2015a). To our knowledge no function for farnesol in the prostate has as yet been suggested. A few papers have been published describing results of the treatment of (prostate and other) cancer with farnesol. Elson et al. (1999) and Mo and Elson (2004) reported that isoprenoid-mediated inhibition of mevalonate synthesis might have potential application in cancer treatment. Statins, which inhibit HMG CoA reductase, reduce the production of both cholesterol and isoprenoids (Wikipedia: Statin). Parikh et al. (2010) reported on statin-induced autophagy by inhibition of geranylgeranyl biosynthesis in prostate cancer PC3 cells. According to Park et al. (2014) farnesol induces apoptosis in DU145 prostate cancer cells through the PI3K/Akt and MAPK pathways. Although some publications report beneficial effects of dietary farnesol on some types of cancer (at 20 g/kg diet on pancreatic cancer: Burke et al., 1997), the limited number of publications on this topic suggests that oncologists, for one reason or another and rightly or wrongly, do not see great potential in the use of farnesol in cancer prevention (Rao et al., 2002) and treatment. We are not aware of any attempts that could have been made in the past decades on the effects of (some of) the 4,000 synthetic compounds with JH activity (Sláma, 2013) for cancer treatment. Many of these compounds are much more active, up to a million times, than farnesol in JH-bioassays. Van Mellaert et al. (1983) reported on the anti-JH effect of some synthetic benzoylphenols that caused sterility in females (inhibition of ovarian development) without being mutagenic like other types of chemosterilants. Whether these compounds were ever tested on their potential as anticancer drugs is unknown. Recently Wilson et al. (2017) reviewed a diverse accessory gland literature which highlights functional analogies between the male reproductive glands of flies and humans and the reasons why the *Drosophila* MAG is a good model for prostate cancer in humans.

### Aging. Alzheimer’s Disease?

In an earlier papers (De Loof et al., 2015a; De Loof, 2017) have suggested that it might be worthwhile to investigate whether a deteriorating mevalonate pathway might somehow be a factor of importance in aging in general and in the initiation of Alzheimer’s disease in particular. Esters of farnesol function as “juvenile hormones” of insects. Apparently, their anti-aging effect is due to the fact that they can keep [Ca^2+^]i low. How this is achieved is largely unknown. An increase in cytoplasmic Ca^2+^ negatively influences the secretion of proteins synthesized by the RER. The main function of the RER is to remove excess Ca^2+^ from the cell by secreting *Ca^2+^-binding proteins*. De Loof (2017) described it as “mechanism 3” in removing excess Ca^2+^ from the cell. Because farnesol is an endogenous blocker of some types of Ca^2+^-channels, a degrading mevalonate biosynthetic pathway will cause an increase in [Ca^2+^]. It may be worthwhile to investigate whether this process might contribute to initiating Alzheimer’s or other major Ca^2+^-dependent diseases.

From many experimental studies in insect development and endocrinology, it is known that farnesol and its JH esters are much more active if topically applied to the cuticle than if injected. In humans, topical application to the skin might be an effective slow release method. If nonetheless injected, they are only effective when mixed with an oil, and if no wetting agents/detergents are added (Wigglesworth, 1969). Oil acts as a slow releasing substance formula. It is also known that not all farnesol/FLS isomers are biologically active. According to Wigglesworth (1969) the all-trans isomeric form of farnesol is the most active. It is this form which is present in the human brain (Roullet et al., 1999). Several thousands of compounds with juvenile hormone activity have been synthesized in recent decades, not with the purpose to develop them as therapeutic drugs in medicine, but as novel growth regulators in insects (Sláma, 2013, 2015). They could all be tested.

## Discussion

Because of the perseverance of Paroulek and Sláma who, half a century after JH was chemically identified, engaged in trying to answer questions with respect to the hormonal status of JH-I in MAGs of *H. cecropia*. Most contemporary researchers, in particular the younger ones, are either not aware that such questions are still open, or think that they are not worth pursuing because this type of research is too dated, or because such projects are currently unfundable from granting agencies. Yet, even to date, in the golden era of molecular biology (genomics and all the omics), some “old-fashioned” experimental approaches, exceptional cases/situations, serendipity, a good historical insight in the development of successive ideas and working hypotheses on the mode of action of JH and ecdysteroids, may ultimately lead to a unifying concept on the endocrinology of the various hormone-dependent aspects of metamorphosis and disease control.

According to Paroulek and Sláma (2014) the accumulation of high amounts of JH-I in MAGs of *H. cecropia* seems to represent an exceptional situation in insects. Thus the fact that many decades ago Carroll Williams used this species for his experimental work on JH may have been a lucky coincidence, not to say a fluke. That does not negate that these results coincidental of “a fluke-like event” had truly major consequences for advancing both insect- and general endocrinology. Indeed, it enabled the chemical identification of juvenile hormone in 1968. Yet, it also left many questions unanswered. Molecular biological research methods have answered some, but not all.

What could be the role of the high concentration of JH-I in MAGS of *H. cecropia*? It may ensure that the sperm cells get a package of farnesol as luggage that helps to keep Ca^2+^ channels, in particular the voltage-gated Ca^2+^ channels (Roullet et al., 1999), closed. It may also be instrumental in keeping Ca^2+^ pumps active during their “swimming journey” to the eggs and, upon fusing of the sperm’s plasma membrane with the membrane of the egg to help pumping out excess Ca^2+^ that entered through the short-lived little wound made by the entering sperm head/nucleus. Thus, after all farnesol may be a means to enable the sperm cell swim and, at the same time protect them against Ca^2+^-induced cell death/apoptosis in the sperm itself as well as in the newly fertilized egg.

The formulation of the duality “endocrine JH versus exocrine JH” by Paroulek and Sláma, represents a fundamental change in the paradigm on the mode of action of JHs and other endogenous farnesol-like sesquiterpenoids. If chemically identical twins are both functional in their physiologic environment, it means that there must exist a common denominator in their mode of action. In other words, some of the receptors of endocrine and exocrine JH must be either the same, or at least be part of a common basic signaling system. In recent years, arguments have been advanced in favor of the idea that this system represents a Ca^2+^-homeostasis system, with numerous factors (De Loof et al., 2014, 2015a,b; De Loof, 2015). The game-changing status of the endocrine-exocrine paradigm also urges for a change in thinking on the “archaeology” of the mevalonate biosynthetic pathway, not only in insects, but in all eukaryotes. Such change has already been initiated in earlier papers (De Loof et al., 2015a). It says that the key role of the mevalonate pathway may not so much be in the biosynthesis of cholesterol (in vertebrates) or of the lipophilic hormone JH (in insects), or of “vertebrate-type steroid hormones.” Rather this role may be that of a (largely overlooked) control system of Ca^2+^-homeostasis, with secondary effects on lipid and steroid biosynthesis. A role as precursor in the mevalonate pathway does not at all exclude an additional role in control of some other physiological process, e.g., Ca^2+^-homeostasis.

An emerging hypothesis: If the role of the tandem farnesol-Ca^2+^-channels was essential for making the flagellum/undulipodium of the ancestral choanoflagellate undulate, could it be that this function continues to be present not only in spermatozoa, but as well in some ciliated somatic cells present in body of animals? Two types of cilia exist: nonmotile or primary cilia which typically serve as sensory organelles, and motile cilia. Primary cilia are specialized multifunctional Ca^2+^-signaling organelles (Delling et al., 2013; Pala et al., 2017). They usually occur one per cell, and nearly all mammalian cells have one. In humans, motile cilia occur in large numbers on the lining of the trachea, and in females, on the lining of the Fallopian tubes where they move the ovum from the ovary to the uterus (Wikipedia: Cilium). This question deserves further exploration.

## Conclusion

The functions of contemporary farnesol/FLS/JHs in the male reproductive system may still reflect evolutionarily ancient roles in the ancestral unicellular progenitor of all animals. A plausible suggestion is that they serve as an overlooked activating “whip to the flagellar undulating beating”, as well as an the overlooked key inhibitor of Ca^2+^-induced apoptosis in sperm cells during their journey from the within the male genital system toward the egg, inside or outside the female, depending on the conditions (dry- or watery environment). Such anti-apoptosis role confirms the view that such role has also been postulated for explaining why the high titre of JH in larvae prevents metamorphosis (De Loof et al., 2015a,b). Finally, the role of farnesol/FLS as hormones is an evolutionarily late acquisition of the mevalonate pathway in animals. Indeed farnesol existed already long before animals came into being.

## Author Contributions

ADL and LS jointly conceived the model and wrote the manuscript.

## Conflict of Interest Statement

The authors declare that the research was conducted in the absence of any commercial or financial relationships that could be construed as a potential conflict of interest.
